# Decaffeination and Neuraminidase Inhibitory Activity of Arabica Green Coffee (*Coffea arabica*) Beans: Chlorogenic Acid as a Potential Bioactive Compound

**DOI:** 10.3390/molecules26113402

**Published:** 2021-06-04

**Authors:** Muchtaridi Muchtaridi, Dwintha Lestari, Nur Kusaira Khairul Ikram, Amirah Mohd Gazzali, Maywan Hariono, Habibah A. Wahab

**Affiliations:** 1 Department of Pharmaceutical Analysis and Medicinal Chemistry, Faculty of Pharmacy, Universitas Padjadjaran, Jl. Bandung-Sumedang KM 21, Jatinangor 45363, Indonesia; 2 Department of Pharmacy, Faculty of Science and Technology, Universitas Muhammadiyah Bandung, Jl. Soekarno-Hatta No. 752, Bandung 40614, Indonesia; dwintha85@gmail.com; 3 Institute of Biological Sciences, Faculty of Science, Universiti Malaya, Kuala Lumpur 50603, Malaysia; nkusaira@um.edu.my; 4 Centre for Research in Biotechnology for Agriculture (CEBAR), Kuala Lumpur 50603, Malaysia; 5 School of Pharmaceutical Sciences, Universiti Sains Malaysia, USM, Penang 11800, Malaysia; amirahmg@usm.my (A.M.G.); habibahw@usm.my (H.A.W.); 6 Faculty of Pharmacy, Campus III, Sanata Dharma University, Paingan, Maguwoharjo, Depok, Sleman, Yogyakarta 55282, Indonesia; mhariono@usd.ac.id

**Keywords:** decaffeination, chlorogenic acid, caffeine, green coffee, HPLC

## Abstract

Coffee has been studied for its health benefits, including prevention of several chronic diseases, such as type 2 diabetes mellitus, cancer, Parkinson’s, and liver diseases. Chlorogenic acid (CGA), an important component in coffee beans, was shown to possess antiviral activity against viruses. However, the presence of caffeine in coffee beans may also cause insomnia and stomach irritation, and increase heart rate and respiration rate. These unwanted effects may be reduced by decaffeination of green bean Arabica coffee (GBAC) by treatment with dichloromethane, followed by solid-phase extraction using methanol. In this study, the caffeine and chlorogenic acid (CGA) level in the coffee bean from three different areas in West Java, before and after decaffeination, was determined and validated using HPLC. The results showed that the levels of caffeine were reduced significantly, with an order as follows: Tasikmalaya (2.28% to 0.097% (97 ppm), Pangalengan (1.57% to 0.049% (495 ppm), and Garut (1.45% to 0.00002% (0.2 ppm). The CGA levels in the GBAC were also reduced as follows: Tasikmalaya (0.54% to 0.001% (118 ppm), Pangalengan (0.97% to 0.0047% (388 ppm)), and Garut (0.81% to 0.029% (282 ppm). The decaffeinated samples were then subjected to the H5N1 neuraminidase (NA) binding assay to determine its bioactivity as an anti-influenza agent. The results show that samples from Tasikmalaya, Pangalengan, and Garut possess NA inhibitory activity with IC_50_ of 69.70, 75.23, and 55.74 μg/mL, respectively. The low level of caffeine with a higher level of CGA correlates with their higher levels of NA inhibitory, as shown in the Garut samples. Therefore, the level of caffeine and CGA influenced the level of NA inhibitory activity. This is supported by the validation of CGA-NA binding interaction via molecular docking and pharmacophore modeling; hence, CGA could potentially serve as a bioactive compound for neuraminidase activity in GBAC.

## 1. Introduction

Indonesia produces at least 10,500.00 bags (60 kg/bags) of coffee per year [1], which contributed to around 6.6% of total coffee production worldwide in 2012 [2]. The consumption of processed coffee-based products in Indonesia increases by approximately 7.5% every year. In addition, Indonesia produces 700 kg of robusta coffee beans/ha/year and 800 kg of Arabica coffee beans/ha/year.

Previous studies have suggested that coffee partially provides health benefits to treat certain diseases, such as type 2 diabetes mellitus [3], besides having antioxidant [4], anti-inflammatory [5], and antibacterial activities [6]. In particular, Van Dam (2005) reported that regular consumption of coffee can reduce the risk of type 2 diabetes mellitus [7], whilst O’Keefe et al. (2013) suggested that it may reduce the risk of death caused by cardiovascular diseases [8].

It is known that coffee contains caffeine at different levels, depending on the seeds, cultivation area, and process. For example, robusta coffee contains 1.7–4.0% of caffeine, which is almost twice the content of Arabica coffee (0.8–1.4%) [9,10,11,12]. Caffeine is reported to cause side effects, such as insomnia, palpitations, an increase in the frequency of urination, headaches, and other symptoms—this is in addition to its main pharmacological effect as a stimulant [13,14,15,16,17].

Decaffeination (decaf) has been well studied as an effort to reduce the adverse effects of caffeine by minimizing its concentration in coffee. This technique is carried out using alcohol or a certain solvent, such as cyclohexane. However, during decaf, the caffeine level in green bean extract of *Coffea arabica,* which is approximately 34.1–38.5 g kg^−1^, is reduced by only 0.4%; hence, decaffeination could not significantly reduce the content of caffeine [18]. However, in general, the chlorogenic acid (CGA) derivatives level in decaffeinated coffee is higher than non-decaf. The 3-chlorogenic acid (3-CGA) and 4-chlorogenic acid (4-CGA) levels in decaffeinated coffee probably increase due to the lixiviation process that occurs during decaf [19]. Interestingly, another study reported that decaf reduced CGA by 10% [20]. The inconsistency of data available in the literature prompted us to conduct this current study, to evaluate the level of chlorogenic acid available in coffee bean extracts before and after the decaffeination process.

Chlorogenic acid is shown to exhibit high activity against the neuraminidase enzyme, which is important in the replication of influenza viruses, such as H5N1 and H1N1 [21]. The intravenous administration of 100 mg/kg/d chlorogenic acid was reported to effectively inhibit H1N1 and H3N2 infections in mice [22]. Based on the reports from previous studies, it is paramount to determine the influence of the decaf process on neuraminidase (NA) inhibition activity as a potentially effective source of anti-influenza in preventing and reducing influenza virus A infection. In this present study, high performance liquid chromatography (HPLC) is used to determine the level of both caffeine and chlorogenic acid in the crude *Coffea arabica* bean extracts, before and after decaffeination. The coffee beans were collected from three different areas in West Java, Indonesia. The coffee samples were then evaluated for their inhibitory effects against the neuraminidase enzyme (NA) of the H5N1 influenza virus.

## 2. Materials and Methods

### 2.1. Plants Material

Crude Arabica coffee (*Coffea arabica* L.) beans were collected from Garut, Pangalengan, and Tasikmalaya in West Java Province, Indonesia, and the specimens were identified in the Laboratory of Plant Taxonomy Herbarium Department of Biology, Faculty of Mathematics and Natural Sciences, University of Padjadjaran (Bandung, Indonesia).

### 2.2. Chemicals and Reagents

Methanol, ethanol, double distilled water, iron (III) chloride, gelatin, ammonia, chloroform, hydrochloric acid, Mayer’s reagent, Dragendorff’s reagent, magnesium, amyl alcohol, ether, vanillin-sulfuric acid reagent, sodium hydroxide, and Liebermann–Burchard reagent were purchased from Merck (Kenilworth, NJ, USA), without further purifications.

### 2.3. For the Enzymatic Inhibition Assay

H5N1 neuraminidase (SINOBIO, Beijing, China), MUNANA [2′2-(4-methylumbelliferyl)-*α*-d-*N*-acetylneuraminic acid sodium salt hydrate] (Sigma, St. Louis, MO, USA), MES [2-(*N*-morpholino) ethanesulfonic acid] (Sigma, St. Louis, MO, USA), and DANA [2,3-didehydro-2- deoxy-*N*-acetylneuraminic acid] (Sigma, St. Louis, MO, USA).

### 2.4. Extraction

Coffee beans (20 g) from Pangalengan, Garut, and Tasikmalaya were pulverized and extracted using the digestion method by soaking and stirring in 250 mL of double distilled water at 40–50 °C for 30 min [23]. The mixture was cooled down at room temperature and then filtered out.

### 2.5. Phytochemical Screening

Phytochemical screening was performed to identify phytochemical substances contained in the crude extract such as alkaloids, flavonoids, tannins, polyphenols, saponins, steroids, and quinone. This screening was carried out based on Farnsworth method [24].

### 2.6. Decaffeination of Coffee Extract

The decaf process was conducted using liquid–liquid extraction by mixing the coffee powder in dichloromethane with a ratio of 1:1. The process was repeated three times and stirred for 10 min at room temperature [25]. Dichloromethane was chosen as the solubility of caffeine; it was higher in this solvent (140 mg/mL) than in water (22 mg/mL) [25].

### 2.7. Determination of Caffeine and Chlorogenic Acid Levels

The cartridge was conditioned sequentially by elution with 6 mL methanol and 3 mL of double distilled water. Moreover, 1 mL of sample was introduced into the cartridge under vacuum and the collected filtrate was re-introduced into the cartridge. Elution was then performed with 10 mL of methanol. This eluate was collected in a clean tube and evaporated to dryness. Following reconstitution of the residue with 10 mL of double distilled water, the sample was then injected into the HPLC system. Reconstituted sample from SPE elution was filtered using Millipore filters 0.45 µm prior to the analysis. The calibration curves for both caffeine and chlorogenic acid were constructed with seven standard concentrations of 1, 5, 10, 25, 50, 100, and 200 µg/mL of the standard solutions. The method development was performed with a HPLC system consisting of an autosampler and an ultraviolet diode array detector. The detector was set at 247 nm and the chromatographic separation was carried out by using Enduro C18 G (250 mm × 4.6 mm, 5 µm). The mobile phase is as follows. For the analysis of caffeine, methanol/water (37:63) and chlorogenic acid, methanol/water (40:60) with 1% acetic acid. The mobile phase was degassed prior to the analysis and delivered at a flow rate of 1.0 mL/min. The method was validated and the linearity, precision, repeatability, recovery, LOD, LOQ, selectivity, and accuracy were determined. The detailed protocol on optimization and validation of this HPLC method was published previously by our team [26].

### 2.8. Neuraminidase Activity

Non-decaf and decaf extract from Tasikmalaya, Garut, and Pangalengan were subjected to the H5N1 neuraminidase assay as neuraminidase inhibitors. Neuraminidase was prepared in 2-(*N*-morpholino) ethanesulfonic acid (MES) buffer (Sigma^®^, St. Louis, MO, USA) to a final concentration of 0.3 U/mL. The substrate MUNANA was prepared in the same buffer to 100 µM. The coffee samples were prepared in 2.5% DMSO (Merck^®^, Kenilworth, NJ, USA) due to its solubility problem that led to difficulty in reaching the desired concentration of between 7.8125 and 125 µg/mL based on the previous studies [27,28]. The times of incubation (shaken in 200 rpm at 37 °C) for the mixture of NA-coffee and NA-coffee-MUNANA were 30 and 60 min, respectively, and the reaction was stopped using glycine before reading. NA inhibitory activity was measured via the fluorogenic substrate, MUNANA excitation at 365 nm and fluorescence emission at 450 using ELISA microplate reader (Tecan-i-control infinite 200Pro) [29]. The data results were analyzed by GraphPad Prism 5.0. The significant difference of IC_50_ between decaf and non-decaf extract for each area was statistically analyzed using *t*-test with GraphPad Prism 4.0 as the software.

## 3. Results and Discussion

### 3.1. Extraction

The extract was obtained from the digestion method as a liquid having a characteristic smell of coffee with green to dark green color. From the 20 g of coffee beans used in the extraction, the quantity of liquid extract obtained from Pangalengan, Garut, and Tasikmalaya beans were 8% (light green), 7.5% (pale green), and 8% (pale green), respectively. 

### 3.2. Phytochemical Screening

Phytochemical screening of extracts from the three different regions showed similar results, in which the extracts contained similar profiles of secondary metabolites (Table 1).

### 3.3. Decaffeination and Determination of Caffeine Using HPLC

Caffeine dissolved better in dichloromethane (140 mg/mL) than in water (22 mg/mL) [24] because it is a relative nonpolar compound. In an equilibrium phase of DCM-water, caffeine would have a higher partition in the nonpolar organic solvent as compared to water. From the SPE process, the analyte was obtained with the yield of 90.7–102.9%, reflecting its capability to dissolve in methanol as well.

**Figure 1:**
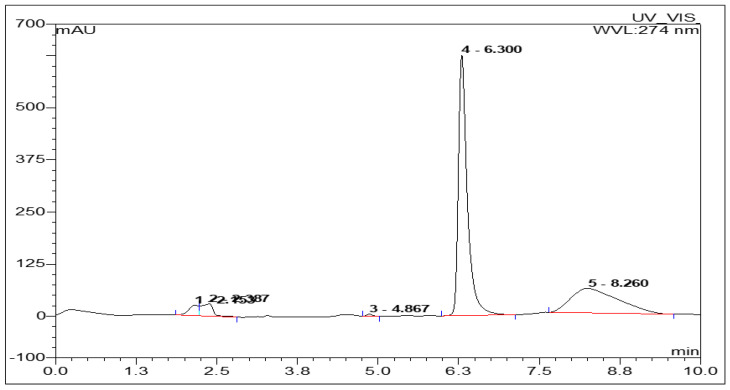
The HPLC chromatogram of caffeine. Enduro C-18G. (250 mm × 4.6 mm) column was used with methanol: water (37:63) mobile phase, the rate 1.0 mL/min and the DAD detector wavelength was set at 274 nm.

It is a generally known fact that the caffeine level in coffee beans is influenced by the species and varieties of the coffee beans, as well as other factors, such as the cultivation environment, period of ripening and storage conditions [30]. In this study, the caffeine levels in three samples of coffee beans obtained from different areas in Indonesia were evaluated. The levels of caffeine in the extracts and the decaf samples were determined via the optimized HPLC method. The best system was chosen based on the retention time (R_t_), lowest plate number (N), highest HETP, lowest tailing factor, and the best capacity factor. Figure 1 shows a typical chromatogram of caffeine, with retention time for caffeine recorded at 6.3 min with a well separated peak, with resolution values > 1.5 [31].

Determination of caffeine using an optimized and validated method of HPLC, and results in the level of caffeine are described in Table 2 and illustrated in Figure 1. The area under the curve (AUC) was generated and applied to the linear regression equation to calculate the concentration of the sample.

The caffeine level in the non-decaffeinated samples from Garut was 1.45%. After decaffeination, the caffeine level decreased to 0.22%. The subsequent decaf processes resulted in a much lower concentration of caffeine, 0.016%, and almost undetectable, 0.00002%, for the second and third processes, respectively.

Similarly, the caffeine content from the coffee beans obtained from Pangalengan reduces after decaffeination from 1.57% to 0.049%. In this sample, the caffeine level was much higher than the sample from Garut after the decaf process.

The coffee beans from Tasikmalaya showed a higher content of caffeine as compared to the other samples. The reduction pattern of caffeine following the decaf process was similar in both samples from Garut and Pangalengan, having the highest caffeine level of 0.097%.

### 3.4. Determination of Chlorogenic Acid Using HPLC

**Figure 2:**
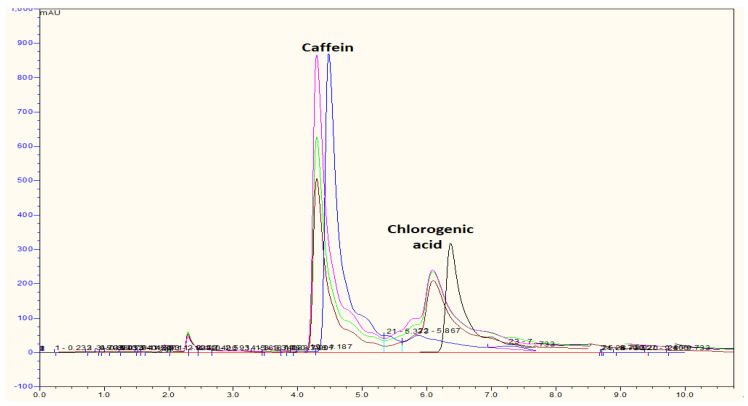
Chromatogram of HPLC (methanol: with acetic acid 1% (40:60) from the extract coffee bean (pink line: sample from Tasikmalaya; brown line: Pangalengan; green line: Garut; blue line: caffeine standard; black line: chlorogenic acid standard).

The 3-caffeoylquinic acid or chlorogenic acid level is analyzed by a mobile phase of methanol: double distilled water in 1% acetic acid (40:60) at a maximum wavelength of 277 nm and a flow rate of 1 mL/min. As shown in Figure 2, both caffeine and chlorogenic acid can be detected in the undecaffeinated Arabica coffee beans by using the HPLC method. The presence of 1% acetic acid is important in the analysis of chlorogenic acid content as it was employed to establish acidic conditions. Li et al. assumed that the addition of acetic acid increases the efficiency of resolution in the separation of peak chromatogram [31].

The extraction process affects the levels of chlorogenic acid in coffee bean extracts. The best extraction method to obtain the highest levels of chlorogenic acid is through Soxhlet extraction with solid phase extraction (SPE) pretreatment and decaffeination [32]. The highest level of chlorogenic acid was detected in the beans obtained from Pangalengan, which was recorded at 0.97%, and this was significantly reduced following decaffeination, as shown in Table 3.

### 3.5. Neuraminidase Activity

Neuraminidase is an enzyme responsible for the cleavage of the glycosidic link of new virions upon budding, which is required for influenza viral replication [33]. Blocking the activity of neuraminidase is considered an effective way to treat influenza. The outbreak of H5N1 in 2005 and the pandemic H1N1 in 2009 indicate the importance of continuous research work to discover new and effective anti-influenza agents to overcome the resistance due to mutations of the virus against the existing neuraminidase inhibitors, such as oseltamivir (Tamiflu^®^) [33].

The bean extracts and decaffeinated samples from Tasikmalaya, Garut, and Pangalengan were further evaluated for their potential as NA inhibitors using MUNANA assay. Table 4 represents the experimental result obtained in comparison to the NA inhibitors as the positive control, i.e., DANA and chlorogenic acid. DANA (2-deoxy, 2,3-dehydro N-acetyl neuraminic acid) is the standard inhibitor used as a positive control in the MUNANA assay. DANA is a sialic acid derivative, which is a natural ligand of neuraminidase [33].

**Figure 3:**
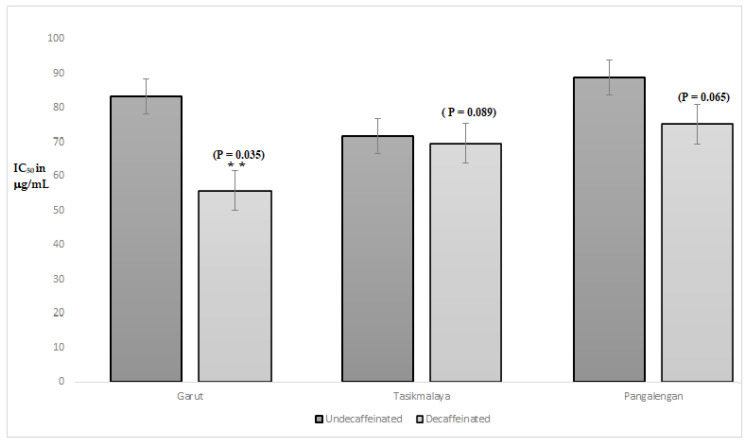
Analysis of statistical tests between uncaffeinated and decaffeinated samples on Neuraminidase activity Statistical analysis was conducted using one sample Student *t*-test (** *p* values < 0.05 were considered significant).

As shown in Table 4, the level of caffeine influences neuraminidase activity of the extracts. Generally, extracts of decaffeinated green bean Arabica coffee from Garut have the lowest level of caffeine than others, in contrast, the neuraminidase inhibition activity is higher than others with IC_50_ 55.74 μg/mL. In Figure 3, there was a significant difference in the inhibitory of neuraminidase activity of the green bean Arabica coffee (GBAC) extract from Garut to the decaffeinated treatment with a *p* value < 0.05 (0.035). Whereas, in the GBAC from Tasikmalaya and Pangalengan, there was, descriptively, a decrease in IC_50_, but statistically using the Student’s *t*-test did not look significant.

Decaffeinated GBAC contains phenolic acid—more than roasted coffee beans [32]; thus, the antioxidant activity of decaffeinated GBAC is higher than the roasted coffee [34,35]. Zhang et al. found that there might be a correlation between antioxidant and anti-influenza activity [36]. The major phenolic acids in GBAC, which has antioxidant activity, are coumaroylquinic acid derivatives as well as a chlorogenic acid (CGA compound) [34]. The results of chlorogenic acid analysis showed that all Arabica coffee beans collected from three locations from different regions in West Java contain caffeine and chlorogenic acid, as shown in Figure 2. Depending on the origin location, the highest caffeine concentrations, respectively, are Tasikmalaya (2.28%) and Pangalengan (1.57%), while the lowest is Garut (1.45%). The highest chlorogenic acids, respectively, are Pangalengan (0.97%) and Garut (0.81%), while the lowest is Tasikmalaya (0.54%). In previous research, Silverolla et al. (2000) reported caffeine in the Ethiopian Arabica coffee bean in the range of 0.46–2.82% [37], while Mohammed et al. (2009) determined caffeine from Iraq in the range of 0.2–2% in *Coffea arabica* [38]. On the other hand, Narita et al. found that the total chlorogenic acid derivatives in Arabica coffee from 22 countries were 3.5–7.5% [39]. Moon et al. analyzed that 3-chlorogenic acid (CGA) in green coffee beans of Arabica were 1.76–3.25% from seven countries [40].

The two compounds evaluated in this study, caffeine and chlorogenic acid, have shown some relation in their activity against NA. Decaffeination reduces the level of caffeine, but at the same time decreases the level of chlorogenic acid in the samples. Chlorogenic acid, which is a type of phenolic acid in coffee bean extracts, can play an important role as an antioxidant, through scavenging the superoxide free radical [41]. This mechanism is an important tool in the development of strategies for keeping organs from failure because of severe complications associated with influenza [41]. Previous studies showed that CGA has an IC_50_ of 44.87 and 62.33 μM against H_1_N_1_ and H_3_N_2_ neuraminidase, respectively [22]. Ding et al. explain that CGA inhibits the influenza A virus as a neuraminidase blocker, whereas Karar et al. predicted that the catechol moiety of CGA might be responsible for neuraminidase inhibition [20]. Herewith, we also study the binding interaction of CGA using molecular docking simulation incorporated with pharmacophore modeling. 

**Figure 4:**
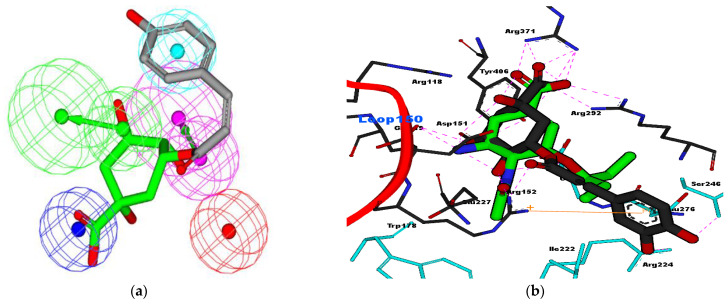
(**a**) CGA fit with oseltamivir pharmacophore features and (**b**) interaction of CGA with NA (PDB ID: 2HU4), compared to oseltamivir (green)-NA. Blue color residues were shown for hydrophobic interaction, while pink dotted lines show hydrogen bond interaction between residues of NA with the ligands. Orange line for cation–*pi* interaction between the aromatic ring of CGA and Arg152 of NA.

In this study, the glycosides of CGA played an important role in the fitting of the pharmacophore model [42]. As shown in Figure 4a, glycoside moiety of CGA fit well into four features of oseltamivir and HipHop2 models [42]. The carboxylic acid of glycoside was fit by the negative ionizable feature for both models, whereas the hydroxyl of glycoside was fit by hydrogen bond donor (HBD) or the hydrogen bond acceptor (HBA) features. The carbonyl ester of CGA also fit into HBA features, whereas the aromatic ring of catechol moiety fit into the hydrophobic feature of the pharmacophore model. Furthermore, the alkyl groups of catechol fit into the hydrophobic feature l, which is similar to isopentyl of oseltamivir (GS4071). Interestingly, the molecular docking analysis of CGA superimposed well with oseltamivir, as shown in Figure 4b.

As described by Luo et al. [43] carboxylic acid of chlorogenic acid binds well with triad arginine of NA, as well as sialic acid. Luo et al. explained that chlorogenic acid is potent for NA of H5N1 (2HU0) because the free energy of binding (FEB) of this compound is lower than oseltamivir. However in this study, the FEB of the oseltamivir tested (−10.99 kcal/mol) was lower than CGA (−9.88 kcal/mol). Cation–*pi* interaction (orange line) might occur between the aromatic ring of catechol moiety and the positively charged Arg152, as shown in Figure 4b. Hydrophobic interaction occurred between the alkyl chain of chlorogenic acid and Ser246, Ile222, and Arg224 at the NA binding site, and the isopentyl of oseltamivir also interacted with the same residues of NA. Unfortunately, due to our limitations, we did not evaluate the synergistic effect of either decaf or non-decaf coffee extract with oseltamivir. However, we will strongly consider this factor in a future study.

## 4. Conclusions

This study showed the ability of SPE and HPLC methods in determining the caffeine levels of both decaffeinated and caffeinated coffee extracts from three different regions in West Java, Indonesia. Among the three regions, Tasikmalaya shows a slightly higher level of caffeine in the samples compared to Garut and Pangalengan. After a repeated decaf process, the caffeine and CGA levels in the three samples showed substantial reduction. Among the three decaf samples, the extract from Garut had the lowest level of caffeine, the highest level of CGA, and the highest inhibition neuraminidase activity—IC_50_ 55.74 μg/mL. Based on this finding, we can conclude that there may be a correlation between the level of caffeine and CGA, which influences the inhibitory activity on NA. The ability of CGA to interact with NA is supported by molecular docking and pharmacophore modeling analysis, and the results suggest the potential of using CGA, as one of the compounds that could be responsible for neuraminidase activity in GBAC.

## Figures and Tables

**Table 1:** Phytochemical screening results.

Secondary Metabolites	Garut	Pangalengan	Tasikmalaya
Alkaloid	+	+	+
Flavonoid	+	+	+
Tannin	-	-	-
Polyphenol	+	+	+
Saponin	-	-	-
Monoterpene and Sesquiterpene	+	+	+
Triterpenoid	+	+	+

Description: (+): detected (-): not detected.

**Table 2:** The caffeine content in coffee bean samples from three districts.

Sample	Average AUC	Concentration (ppm)	% Level
Coffee Bean Samples from Garut			
Non decaffeinated	77,794 ± 117	1163.56 ± 541.11	1.45 ± 0.05
decaffeinated one times	14,293 ± 26	175.38 ± 374.46	0.22 ± 0.008
decaffeinated two times	3829 ± 45	12.54 ± 042.32	0.016 ± 0.004
decaffeinated three times	3025 ± 22	0.016 ± 0.005.52	0.00002 ± 0.00
Coffee Bean Samples from Pangalengan			
Not decaffeinated	83,924 ± 136.67	1258.95 ± 45.21	1.57 ± 0.88
decaffeinated one times	19,598 ± 177.48	257.92 ± 67.02	0.32 ± 0.05
decaffeinated two times	8686 ± 660.96	88.12 ± 14.33	0.11 ± 0.02
decaffeinated three times	55,716 ± 150.03	39.64 ± 11.54	0.049 ± 0.01
Coffee Bean Samples from Tasikmalaya			
Not decaffeinated	120,244 ± 115.45	1824.16 ± 32.22	2.28 ± 0.05
decaffeinated one times	26,606 ± 98.20	366.98 ± 43.14	0.46 ± 0.01
decaffeinated two times	8292 ± 56.78	81.98 ± 13.08	0.10 ± 0.01
decaffeinated three times	3523 ± 45.67	7.77 ± 09.01	0.097 ± 0.00

**Table 3:** Level of chlorogenic acid in coffee bean samples.

	Chlorogenic Acid Level (ppm)		% Chlorogenic Acid	
Sample *	Undecaffeinated	Decaffeinated 3×	Undecaffeinated	Decaffeinated 3×
Garut	565.95 ± 7.35	282.20 ± 2.01	0.81 ± 0.035	0.029 ± 0.01
Pangalengan	659.46 ± 29.41	388.35 ± 13.04	0.97 ± 0.064	0.005 ± 0.00
Tasikmalaya	741.23 ± 58.83	118.22 ± 3.77	0.54 ± 0.021	0.001 ± 0.00

* n = 3.

**Table 4:** The NA inhibition activities of non-decaf and decaf coffee from different regions in West Java.

Treatments	% Enzyme Inhibition	IC_50_ (μg/mL)	Level of Caffeine (%)	Level of Chlorogenic Acid
Blank	0	0	-	
DANA ^a^	90.01 ± 24.55	5.93 ± 0.54	-	
Garut extract	72.26 ± 22.02	83.80 ± 12.38	1.45 ± 0.05	0.81 ± 0.035
Tasikmalaya extract	83.28 ± 18.90	71.70 ± 16.03	2.28 ± 0.05	0.54 ± 0.021
Pangalengan extract	79.90 ± 18.43	88.79 ± 22.11	1.57 ± 0.88	0.97 ± 0.06
Garut decaf	80.84 ± 25.01	55.74 ± 08.89 ^b^	0.00002 ± 0.00	0.029 ± 0.01
Tasikmalaya decaf	88.89 ± 17.34	69.70 ± 18.23	0.097 ± 0.00	0.001 ± 0.00
Pangalengan decaf	80.00 ± 18.23	75.23 ± 19.02	0.049 ± 0.01	0.005 ± 0.00
CGA ^c^	98.51 ± 15.34	± 10.55	-	

^a^ DANA: (2-deoxy, 2,3-dehydro N-acetyl neuraminic acid). ^b^ *p* values < 0.05 were considered significant. ^c^ CGA: chlorogenic acid.

## Data Availability

Not report any data.
